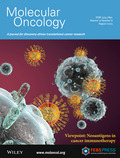# Issue Information

**DOI:** 10.1002/1878-0261.13238

**Published:** 2023-08-03

## Abstract

Deciphering the cancer peptide landscape, as well as delineating neopeptide properties required to induce a potent immune response is needed to optimise the efficacy of targeting neopeptides via cancer vaccines. Read the full Viewpoint article by Yochai Wolf and Yardena Sameuls in pp. 1457–1459.